# Healthcare worker burnout during a persistent crisis: a case–control study

**DOI:** 10.1093/occmed/kqae032

**Published:** 2024-05-13

**Authors:** S Appelbom, A Nordström, A Finnes, R K Wicksell, A Bujacz

**Affiliations:** Department of Learning, Informatics, Management and Ethics, Karolinska Institutet, Stockholm, Sweden; Department of Learning, Informatics, Management and Ethics, Karolinska Institutet, Stockholm, Sweden; Department of Clinical Neuroscience, Karolinska Institutet, Stockholm, Sweden; Department of Clinical Neuroscience, Karolinska Institutet, Stockholm, Sweden; Pain Clinic, Capio St Göran Hospital, Stockholm, Sweden; Department of Learning, Informatics, Management and Ethics, Karolinska Institutet, Stockholm, Sweden

## Abstract

**Background:**

During the immediate outbreak of the COVID-19 pandemic, burnout symptoms increased among healthcare workers. Knowledge is needed on how early symptoms developed during the persistent crisis that followed the first pandemic wave.

**Aims:**

To investigate if high levels of burnout symptoms during the first pandemic wave led to high burnout and depressive symptoms up to a year later, and if participation in psychological support was related to lower levels of symptoms.

**Methods:**

A longitudinal case–control study followed 581 healthcare workers from two Swedish hospitals. Survey data were collected with a baseline in May 2020 and three follow-up assessments until September 2021. The case group was participants reporting high burnout symptoms at baseline. Logistic regression analyses were performed separately at three follow-ups with case–control group assignment as the main predictor and burnout and depression symptoms as outcomes, controlling for frontline work, changes in work tasks and psychological support participation.

**Results:**

One out of five healthcare workers reported high burnout symptoms at baseline. The case group was more likely to have high burnout and depressive symptoms at all follow-ups. Participation in psychological support was unrelated to decreased burnout and depressive symptoms at any of the follow-ups.

**Conclusions:**

During a persistent crisis, healthcare organizations should be mindful of psychological reactions among staff and who they place in frontline work early in the crisis. To better prepare for future healthcare crises, preventive measures on burnout are needed, both at workplaces and as part of the curricula in medical and nursing education.

## Teaser

The results from this study, investigating burnout symptoms among healthcare workers during a persistent crisis, showed that healthcare workers who reported high burnout symptoms in the beginning of the COVID-19 pandemic were more likely to experience high burnout symptoms up to a year later. Repeated periods of frontline work had a limited impact on burnout rates. Instead, the reporting of burnout symptoms was related to the work environment in the beginning of the pandemic.

**Key learning points**

**What is already known about this subject:**

- Increased prevalence of burnout symptoms has been reported among healthcare workers during the COVID-19 pandemic.
- For a sub-group of healthcare workers with higher symptoms, burnout rates were elevated also later in the crisis.
- It is not known whether burnout and depressive symptoms later in the pandemic were predicted mainly by the early burnout symptoms or the recurring periods of frontline work for this group of healthcare workers.

**What this study adds:**

- High burnout symptoms in response to the work environment during the first pandemic wave were the main predictor of both high burnout and depressive symptoms during later periods of the crisis, regardless of the intensity of frontline work or participation in psychological support.

**What impact this may have on practice or policy:**

- When forming policies on how to respond to future crises in healthcare, healthcare workers’ psychological reactions to the work environment during the initial stages of the crisis should be a key focus.
- If the crisis persists over a longer period, healthcare organizations may benefit from being extra mindful of the work environment of staff members who showed signs of burnout right before, or early in the crisis.

## INTRODUCTION

During the immediate outbreak of the COVID-19 pandemic, healthcare workers faced high emotional and physical demands at the same time as resources to cope with them were limited [1,2]. The prevalence of burnout symptoms also increased among healthcare workers during this period with pooled prevalence between 34% and 52% [3,4]. In the ICD-11, the World Health Organization defines burnout as ‘a syndrome conceptualized as resulting from chronic workplace stress that has not been successfully managed’ [5]. More specifically, burnout refers to the combination of high job demands and insufficient job resources that implicates the workers’ ability to cope with those demands [6,7]. When faced with this imbalance, the worker experiences symptoms of emotional exhaustion and depersonalization from work [8]. Although burnout is an occupational phenomenon [5], conflicting demands in personal life can also lead to burnout symptoms [9].

Even before the COVID-19 pandemic, healthcare workers had increased risks of burnout. In 2020, pre-pandemic burnout prevalence of 12% among early-career nurses in Sweden [10], and 10% among nurses in Europe and Central Asia [11] was reported.

Although burnout is not a clinical condition, a long-term imbalance between demands and resources is related to higher risk of sickness absence [12]. Still, experiencing high burnout symptoms during a shorter period of a demanding work environment does not necessarily mean that the worker will face symptoms long term [13]. Several studies on burnout trajectories among healthcare workers during the COVID-19 pandemic reported elevated burnout symptoms during the first outbreak of the COVID-19 pandemic, which would later decrease [14–17].

However, for a sub-group of healthcare workers with strong stress reactions during the first pandemic wave, the high symptom levels were persistent also during later periods of the COVID-19 pandemic [14,18–20]. A possible reason for this may be that their strong reaction to the immediate crisis during the pandemic outbreak led to an elevated risk of later psychological strain in terms of both depressive and burnout-related symptoms [10,13,21,22]. This group of healthcare workers may, therefore, have been more vulnerable to changes in the work environment during the recurring periods of increased patient intake later in the pandemic [15].

To the best of our knowledge, only one study has explicitly studied the relationship between early onset of burnout symptoms and the risk of later strain among healthcare workers during the COVID-19 pandemic. The results showed that burnout symptoms during the first pandemic wave predicted about 37% of burnout symptoms among US frontline healthcare workers during the second wave. However, it was not clear how job demands related to the treatment of COVID-19 patients impacted the burnout rates [20]. More knowledge is needed on the persistence of burnout and depressive symptoms during different stages of a long-term crisis in relation to early onset of burnout rates and changes in the work environment, both between and during consecutive pandemic waves [23].

Occupational health services can support workplaces in the implementation of organizational changes that prevent burnout by fostering a healthy work environment [24]. However, during a crisis, demands are high, and the resources limited, so health-promoting interventions should rather focus on secondary interventions that help healthcare workers cope with the crisis [24]. During the COVID-19 pandemic, many healthcare organizations implemented psychological support with the aim to limit psychological stress reactions, such as burnout and depressive symptoms, caused by the demanding work environment [25–27].

The aim of the present study was to (i) describe the prevalence of burnout and depressive symptoms among healthcare workers up to a year after the onset of the COVID-19 pandemic, (ii) examine if high levels of burnout symptoms early in the pandemic led to high burnout and depressive symptoms up to a year later, and (iii) additionally examine if those who participated in psychological support initiatives displayed lower levels of burnout or depressive symptoms across the first year of the COVID-19 pandemic.

## METHODS

Healthcare workers from two hospitals in the Stockholm Region, Sweden, were invited to participate in a longitudinal survey during the COVID-19 pandemic. Data were collected at four timepoints with baseline in May and June 2020, and follow-ups in September 2020, February 2021 and June 2021. Compared to the timeline of the COVID-19 pandemic in Sweden, the surveys coincided with the first pandemic wave (baseline), between the first and second waves (follow-up 1), the overlap between the second and third waves (follow-up2), and after the third wave (follow-up 3) [28]. The study was reviewed and approved by the Swedish Ethical Review authority (reference numbers 2020-01795, 2020-03495, 2020-04959, 2020-06602, 2022-01546-02). The participants involved provided written informed consent before answering the baseline survey.

**Figure 1.**
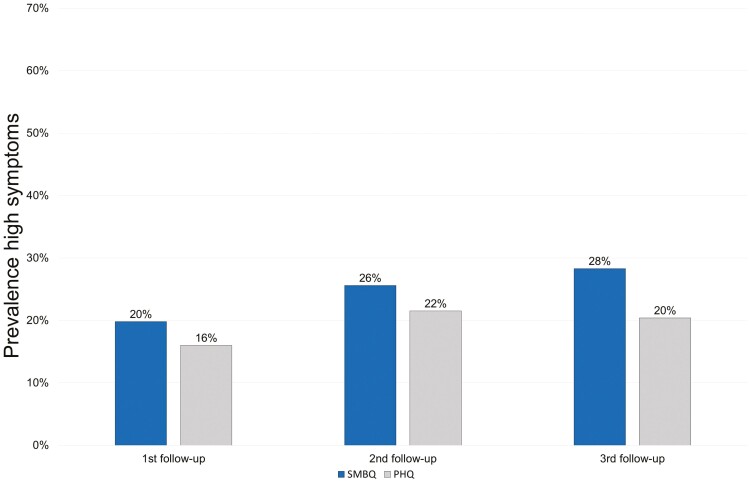
Prevalence of burnout (SMBQ) and depressive symptoms (PHQ) at follow-ups.

A case–control design was applied to investigate the association between high burnout symptoms during the early stages of the COVID-19 pandemic and high symptoms throughout the first year of the pandemic. Out of 2262 invited, 681 healthcare workers were enrolled in the study (see Figure 1, available as Supplementary data at *Occupational Medicine* Online). Participants (*n* = 581) who answered the burnout questionnaire at baseline were included in the analytic sample and assigned to the case and control groups based on high or low burnout symptoms at baseline.

Case and control groups were created based on the level of work-related burnout symptoms measured using the seven-item version of the Oldenburg Burnout Inventory (OLBI). Items were rated on a 4-point scale, ranging from 1 = ‘Not accurate at all’ to 4 = ‘Totally accurate’. A cut-off mean score of 3 or higher was considered a high level of burnout symptoms. The version used in the present study was validated in a Swedish healthcare context, and the cut-off values were established for nurses as the norm group [7]. To limit missing data, mean values were calculated for all participants who answered at least six out of seven items. Scale means were 2.39, 2.20, 2.35 and 2.41 for baseline and each follow-up, respectively. The scale reliability was high at all timepoints (see Table 4, available as Supplementary data at *Occupational Medicine* Online).

At follow-ups, burnout symptoms were measured with the Shirom-Melamed Burnout Questionnaire (SMBQ). SMBQ is a validated screening tool for detecting clinical symptom levels of burnout within a general population in Sweden [29]. For this study, a six-item version was used, measuring emotional and physical exhaustion, as well as cognitive weariness [30]. Items were rated on a 7-point scale, ranging from 1 = ‘Almost never’ to 7 = ‘Almost always’. Cut-off values were chosen based on pre-pandemic data from the general population in Sweden, where the 75% percentile corresponded to an average of 4 for women and 3.5 for men on a 7-point scale [30]. A mean value of 4 was chosen as a cut-off for high burnout symptoms in the present study. A mean value was calculated for all participants who answered at least five out of six items. Scale means were 2.68, 2.97 and 3.02 for each follow-up, respectively. The scale reliability was high for all time points (see Table 4, available as Supplementary data at *Occupational Medicine* Online).

Depressive symptoms were measured using the two-item screening version of the Patient Health Questionnaire (PHQ-2). PHQ-2 measures symptoms of anhedonia and low mood within a 2-week period [31]. Items were rated on a 4-point scale ranging from 0 = ‘Not at all’ to 3 = ‘Nearly every day’. A cut-off value of 1.5 or higher on the mean scale was considered an indicator of high levels of depressive symptoms [31,32]. Due to high correlations between the two items, a mean value was calculated for all participants who answered at least one out of the two items. Scale means were 0.60, 0.58, 0.76 and 0.69 from baseline and each follow-up, respectively (see Table 5, available as Supplementary data at *Occupational Medicine* Online).

Frontline work was recoded based on whether participants were frequently exposed to COVID-19 due to their work at frontline with COVID-19 patients. Participants indicating that they had treated COVID-19 patients within the last 2 weeks on several occasions or daily were coded as 1 = Frontline workers. Participants who had not treated COVID-19 patients or had treated them on one occasion were coded as 0 = non-frontline workers.

Participants stated whether their work tasks had changed due to the COVID-19 pandemic with a single item coded as 1 = Yes, and 0 = No. In the baseline survey, changed tasks since the beginning of the COVID-19 pandemic were noted while follow-ups measured changes since the previous survey.

Participants rated their level of participation in seven different psychological support types, using a single item: ‘Have you been offered any of the following types of support during the current pandemic?’, with 1 = ‘No’, 2 = ‘Yes, I have been offered but not used or participated in them’, 3 = ‘Yes, I have occasionally used or participated in them’, and 4 = ‘Yes, I have used them or participated in them on several occasions’ as response options. The response options were dichotomized, with options 1 and 2 coded as having not participated, and options 3 and 4 coded as having participated. The support types were categorized into two variables: *passive* and *active* psychological support, presented in Table 1. Participation in each category (active/passive) was noted if participants had indicated participation in at least one support type belonging to that category.

**Table 1.** Description of types of psychological support measured in the surveys

Support variable	Type of support
Passive support	Access to a quiet space, for example, a staff room to rest or recover
	Access to information on stress management or mental health
	Education or other training regarding the COVID-19 disease or personal protective equipment
	Education or other training in potentially traumatic situations at work
Active support	Scheduled appointments to meet with colleagues to check in on or discuss how they feel
	Collegial support interventions, for example, peer support or mentorships
	Scheduled group sessions lead by psychologists, counsellors, priests, HR or others
	Individual conversations led by psychologists, counsellors, priests, HR or others

Prevalence of burnout at baseline and at each of the three follow-ups was estimated for the entire sample. The association between high burnout levels at baseline and symptoms at each follow-up was analysed using logistic regression, with case and control group membership as the main predictor and cut-off scores for high levels of burnout and depressive symptoms as outcome measures (Model 1). Second, the model was extended by controlling for frontline work and change of work tasks (Model 2), and psychological support participation (Model 3). Analyses were conducted for each outcome separately. All statistical analyses were conducted using Jamovi version 2.2 [33].

## RESULTS

Baseline sample characteristics and differences between the case (*n* = 119) and control (*n* = 462) groups are presented in Table 2. Of the 581 participants enrolled at baseline, 436 (75%) completed the first follow-up, 385 (66%) the second and 329 (57%) the third. See Supplementary Materials for more details on dropouts. The overall number of responses on burnout (SMBQ) at follow-ups 1, 2 and 3 were 414, 352 and 300, respectively. For depressive symptoms, the number of responses were 405, 339 and 294 for follow-up 1, 2 and 3, respectively. Proportions of participants engaged in frontline work, with changed work tasks, and psychological support participation at follow-ups are outlined in Table 6 (available as Supplementary data at *Occupational Medicine* Online).

**Table 2.** Characteristics of the analytic sample

Characteristics	Analytic sample		Case group		Control group		*t*(193)	χ^2^	*df* (*n*)	*P*-value
	*M* (SD)	*n* (%)	*M* (SD)	*n* (%)	*M* (SD)	*n* (%)				
Age	45 (11.3)		41 (10.4)		46 (11.3)		3.98			<0.001
Women		455 (79)		88 (76)		367 (80)		0.781	1 (577)	>0.05
Occupation								30.3	3 (577)	<0.001
Nurse		218 (38)		68 (58)		150 (33)				
Assistant nurse		126 (22)		24 (20)		102 (22)				
Physician		102 (18)		16 (14)		86 (19)				
Non-medical		131 (23)		10 (9)		121 (26)				
Previous treatment mental illness								14.7	2 (576)	<0.001
No treatment		449 (78)		77 (65)		372 (81)				
More than 3 months prior		104 (18)		32 (27)		72 (16)				
Within 3 months		23 (4)		9 (8)		14 (3)				
Frontline work		313 (62)		98 (85)		215 (55)		33.5	1 (509)	<0.001
Changed tasks		309 (54)		77 (65)		232 (51)		7.26	1 (575)	<0.01
Passive support		442 (78)		94 (81)		348 (78)		0.612	1 (564)	>0.05
Active support		316 (56)		84 (74)		232 (52)		17.7	1 (562)	<0.001

*Notes*: Comparison groups were men, non-frontline workers, unchanged work tasks, and not having participated in passive/active support.

At baseline, 119 (21%) participants reported high burnout symptoms (i.e. the case group). The burnout prevalence during each follow-up was then estimated using SMBQ scores, presented in Figure 1.

**Figure 2.**
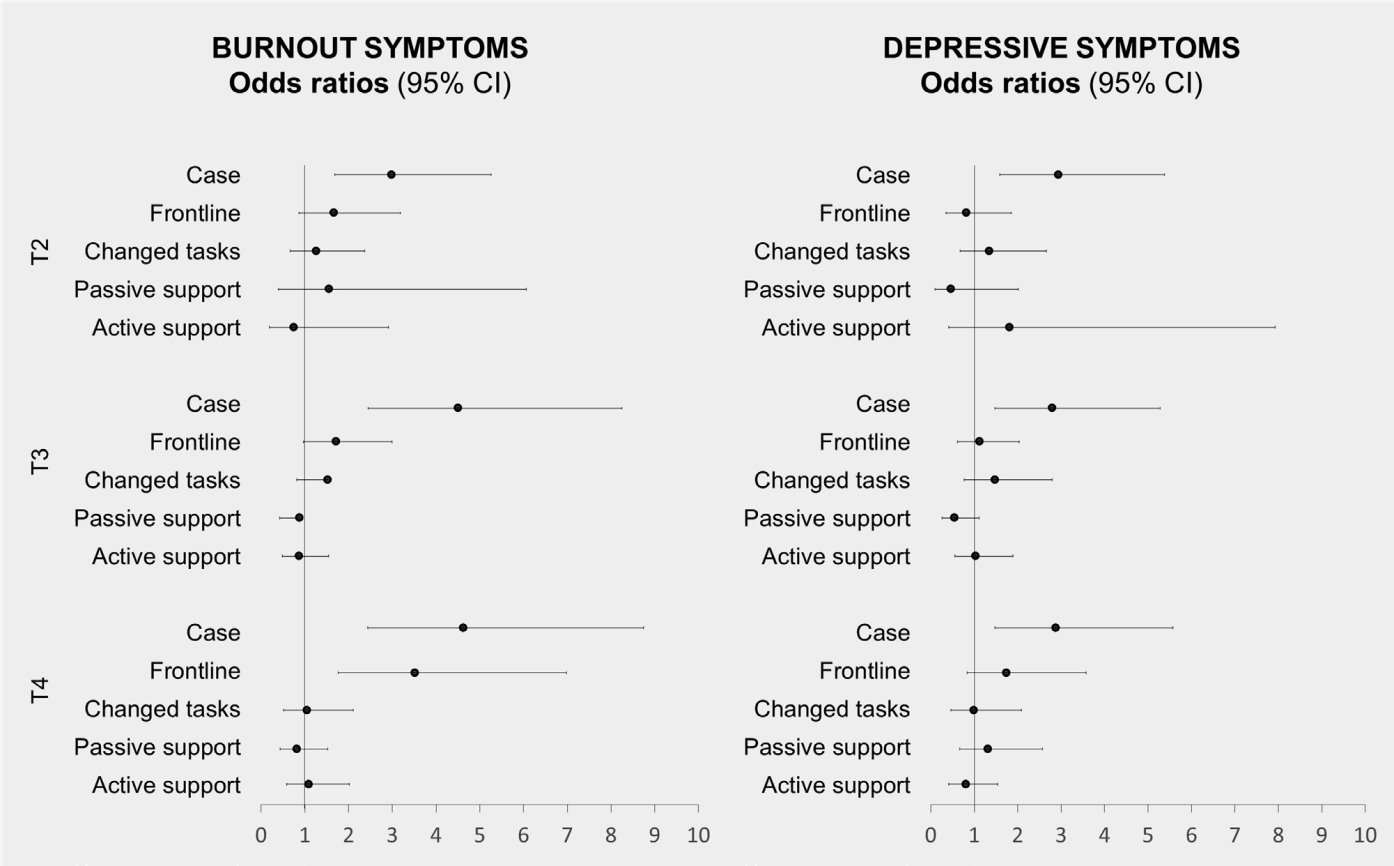
Odds ratios and 95% CI for each predictor in Model 3 and for each follow-up, respectively (T2 to T4).

Figure 2 displays the logistic regression results for each outcome separately. At follow-up 1, cases were more likely to have high burnout symptoms compared to the controls (odds ratio [OR] = 3.061, 95% confidence interval [CI]: 1.798–5.211, *P* < 0.001, Model 1). The effect remained both at follow-up 2 (OR = 5.018, 95% CI: 2.868–8.780, *P* < 0.001, Model 1) and follow-up 3 (OR = 4.874, 95% CI: 2.699–8.802, *P* < 0.001, Model 1).

Across all follow-ups, the association was unchanged also after controlling for frontline work and change of work tasks in Model 2 (follow-up 1 OR = 3.120, 95% CI: 1.774–5.489, *P* < 0.001; follow-up 2 OR = 4.594, 95% CI: 2.593–8.140, *P* < 0.001; follow-up 3 OR = 4.434, 95% CI: 2.383–8.250, *P* < 0.001), as well as psychological support participation in Model 3 (follow-up 1 OR = 2.979, 95% CI: 1.687–5.260, *P* < 0.001; follow-up 2 OR = 4.496, 95% CI: 2.451–8.249; follow-up 3 OR = 4.622, 95% CI: 2.443–8.746, *P* < 0.001).

At the third follow-up, participants categorized as frontline workers were also more likely to report high burnout symptoms. This was the case in both Model 2 (OR = 3.357, 95% CI: 1.741–6.473, *P* < 0.001), and after adding the psychological support in Model 3 (OR = 3.511, 95% CI: 1.765–6.983, *P* < 0.001). No other predictor had a statistically significant association with burnout symptoms (see Figure 2). A more detailed presentation of all model results is presented in Tables 7–9 (available as Supplementary data at *Occupational Medicine* Online).

Cases were also more likely to have high depressive symptoms later in the pandemic. The effect was similar at follow-up 1 (Model 1 OR = 3.175, 95% CI: 1.795–5.615, *P* < 0.001), follow-up 2 (Model 1 OR = 3.415, 95% CI: 1.917–6.083, *P* < 0.01), and follow-up 3 (Model 1 OR = 2.808, 95% CI: 1.502–5.250, *P* < 0.01).

This effect remained unchanged also after controlling for frontline work and change of work tasks in Model 2 (follow-up 1 OR = 3.051, 95% CI: 1.669–5.578, *P* < 0.001; follow-up 2 OR = 3.086, 95% CI: 1.710–5.570, *P* < 0.001; follow-up 3 OR = 2.657, 95% CI: 1.388–5.088, *P* < 0.01), and after adding psychological support in Model 3 (follow-up 1 OR = 2.927, 95% CI: 1.591–5.384, *P* < 0.001; follow-up 2 OR = 2.794, 95% CI: 1.478–5.280, *P* < 0.01; follow-up 3 OR = 2.872, 95% CI: 1.481–5.569, *P* < 0.01). No other predictor had a statistically significant association with depressive symptoms (see Figure 2). See Tables 10–12 for details of all model results (available as Supplementary data at *Occupational Medicine* Online).

## DISCUSSION

The results of this study showed that one out of five healthcare workers experienced high burnout symptoms during the first wave of the COVID-19 pandemic. Affected healthcare workers were also more likely to experience both high burnout and depressive symptoms during follow-ups, compared to those with low burnout symptoms at baseline. Participation in psychological support was unrelated to the level of reported symptoms at follow-ups.

One strength of this study is the longitudinal design which allowed us to measure burnout symptoms over the course of a persistent crisis. We were also able to capture changes in the work environment over the course of the COVID-19 pandemic. By using burnout instruments validated within a Swedish healthcare context, we were able to compare the burnout prevalence with pre-pandemic levels and generalize our findings to Swedish healthcare workers.

The main limitation of our study is that we lack pre-pandemic data for our sample. It was more common among cases to have a previous history of mental illness and we do not know to what extent previous health problems overshadow the COVID-19-related effects on burnout. Another limitation was the use of self-reported data, which introduces the risk of biases (e.g. social desirability or inaccurate recollections). The low inclusion rate (26%) also introduces the risk of selection bias. Regarding our statistical analysis, the use of a single model might have led to a more straightforward conclusion, but our analytical approach enables a closer perspective on the specific issue of the persistence of high symptoms at different stages of a prolonged crisis.

At baseline, the prevalence of high burnout symptoms was 21%. Which is a considerable increase compared to the reported pre-pandemic prevalence of 10% among nurses in Sweden [10], and 12% among nurses in Europe and Central Asia [11]. This indicates that, like other countries [34], the COVID-19 pandemic negatively impacted the psychological well-being among healthcare workers in Sweden.

Experiencing high burnout symptoms during the first wave was the main predictor of high symptoms of both burnout and depression later in the pandemic. Building on the already existing literature on the long-term consequences of burnout [10,22,35], this finding implies that the early onset of symptoms is the main risk factor for continuing symptoms also during a persistent crisis.

When comparing the case to control group at baseline, we found important differences. Known risk factors related to burnout such as being a nurse, work in the frontline and have changed work tasks were more common in the case group [16,17,36–38]. It was also more common in the case group to have sought help for mental health issues. This group was therefore likely more vulnerable to the increased demands in the early pandemic [21].

Interestingly, at the last time point, over a year after the pandemic outbreak, frontline work increased the risk of burnout. This suggests that, over the course of a persistent crisis, there may be an added burden from being re-exposed to high job demands that increase the risk of both burnout and depressive symptoms [15].

Across all follow-ups, psychological support participation was not related to the likelihood of experiencing high symptoms. However, it was more common among the case group to use the active psychological support at baseline. Because of the strong relationship between case group membership and symptoms, the association between psychological support and stress symptoms may have been difficult to capture within the context of this study. Also, due to lack of pre-pandemic data, the effect of the interventions was not possible to assess.

An effect of psychological support on stress reactions during healthcare crises has been difficult to establish in many studies [23,27]. Future research should use more objective measurements such as participation logs and, if possible, pre-crisis data for comparison. Rather than measuring the changes in symptoms, it might be beneficial to investigate changes in resources that help healthcare workers cope better [39]. Such a resource could be perceived social support, a resource known to mitigate burnout symptoms when the demands are high [40].

During a persistent crisis, healthcare workers’ responses to the strained work environment in the immediate outbreak is a major risk factor for developing high burnout and depressive symptoms later in the crisis. A strained work environment may be difficult to avoid, but, if possible, healthcare organizations should be mindful of who they place in the frontline. Especially among those healthcare workers with a strong initial burnout reaction. Future research should address how and when changes in the work environment translate into burnout and depressive symptoms over time during a persistent crisis. Occupational health researchers should focus on identifying individual-centred interventions [24] that in a feasible way support healthcare workers experiencing strong stress reactions during an ongoing crisis.

## Supplementary Material

kqae032_suppl_Supplementary_Material

## Data Availability

Raw data will not be shared due to the constraints in the information included in the written consent.
