# Supplementary material for: Healthcare worker burnout during a persistent crisis: a case–control study
Source: Occup Med (Lond). 2024 May 13;74(4):297–303. doi: 10.1093/occmed/kqae032 (PMC11165371; doi:10.1093/occmed/kqae032)
Supplement: kqae032_suppl_Supplementary_Material [file kqae032_suppl_supplementary_material.docx]

**Supplementary materials**

[DESCRIPTION OF COHORTS 2](#_Toc159926571)

[DROPOUT ANALYSIS AND MISSING VALUES 3](#_Toc159926572)

[RELIABILITY ANALYSIS 7](#_Toc159926573)

[RESPONSES ON SECONDARY PREDICTORS 8](#_Toc159926574)

[LOGISTIC REGRESSION MODELS 9](#_Toc159926575)

## DESCRIPTION OF COHORTS

Participants were invited from two different hospitals in the Stockholm Region, referred to as Site 1 and Site 2. At Site 1, 473 staff members working within intensive care and acute medicine were invited to participate in the study. At Site 2, all (1789) staff members currently affiliated or recently employed at the hospital were invited to participate in the study. Based on actual employee rates in 2019, the number of survey responses from Site 2 roughly corresponds to 40% of all hospital staff (1). See Figure S1 for more details on recruitment.

**Figure S1**

Flowchart over the recruitment process.

Invited to participate
*n* = 2262

Excluded, *n* = 1581
Reason: Did not provide written consent, other active decline of participation or did not respond to baseline survey

Recruited participants
*n* = 681

Excluded, *n* = 100
Reason: No baseline burnout score

Analytic sample
*n* = 581

Control group
*n* = 462

Case group
*n* = 119

## DROPOUT ANALYSIS AND MISSING VALUES

At baseline, data were missing in 0.2-1.4% across the OLBI items. At the first follow-up, data were missing in 5-5.7% across the SMBQ items, and in 7.8% across the PHQ-2 items. At the second follow-up, data were missing in 8.3-9.6% across the SMBQ items, and in 12.5% across the PHQ-2 items. At the third follow-up, data were missing in 8.5-9.7% across the SMBQ items, and in 10.9% across the PHQ-2 items.

The percentage of participants with one item missing was: 4.5% for OLBI at baseline, 9.2% for SMBQ and 8.5% for PHQ-2 at follow-up 1, 12.2% for SMBQ and 13% for PHQ-2 at follow-up 2, and 8.8% for SMBQ and 8.9% for PHQ-2 at follow-up 3.

Dropout analysis was performed by comparing the responders to non-responders at each follow-up on case/control grouping, hospital site, gender, age, occupation, tenure, mental health history, frontline work, changes in work tasks, and participation in psychological support.

There were a few differences between dropouts and non-dropouts (Table S1-S3): The non-dropouts had a higher mean age and were more experienced within their professions at all follow-ups. At the first follow-up, non-dropouts worked at one of the hospital sites, with only intensive care- and acute medicine units participating in the study, to a greater extent. Further, it was more common among non-dropouts to have participated in active forms of support at this time-point. At the third follow-up, there was a larger proportion of physicians and fewer assistant nurses among the non-dropouts. Also, the dropouts had been given new work tasks during the first three months of the pandemic to a larger extent compared with non-dropouts in the final follow-up.

**Table S1**

Drop-out analysis at the first follow-up.

|  | Non-dropouts | Dropouts | *t* | χ^2^ | *df* | *p* |
| --- | --- | --- | --- | --- | --- | --- |
| Age, *M* (*SD*) | 45.7(10.8) | 42.3 (12.3) | -2.94 |  | 220 | .002 |
| Group, *n* (%) |  |  |  | 0.0955 | 1 (581) | .757 |
| Case | 88 (20%) | 31 (21%) |  |  |  |  |
| Control | 348 (80%) | 114 (79%) |  |  |  |  |
| Hospital site, *n* (%) |  |  |  | 18.6 | 1 (581) | <.001 |
| Site 1 | 166 (38%) | 27 (19%) |  |  |  |  |
| Site 2 | 270 (62%) | 118 (81%) |  |  |  |  |
| Gender, *n* (%) |  |  |  | 1.53 | 1 (577) | .216 |
| Women | 337 (78%) | 118 (82.5%) |  |  |  |  |
| Men | 97 (22%) | 25 (17.5%) |  |  |  |  |
| Occupation, *n* (%) |  |  |  | 1.11 | 3 (577) | .774 |
| Assistant Nurse | 93 (21%) | 33 (23%) |  |  |  |  |
| Nurse | 161 (37%) | 57 (30%) |  |  |  |  |
| Physician | 79 (18%) | 23 (16%) |  |  |  |  |
| Non-clinical | 102 (23%) | 29 (20%) |  |  |  |  |
| Tenure *n* (%) |  |  |  | 24.9 | 3 (564) | .001 |
| 0-1 years | 9 (2%) | 12 (9%) |  |  |  |  |
| 2-5 years | 55 (13%) | 34 (24%) |  |  |  |  |
| 6-10 years | 69 (16%) | 23 (16%) |  |  |  |  |
| 11+ years | 290 (69%) | 72 (51%) |  |  |  |  |
| Mental health history at inclusion, *n* (%) |  |  |  | 0.0575 | 2 (576) | .972 |
| No history | 37 (78%) | 112 (78%) |  |  |  |  |
| Last 3 months | 17 (4%) | 6 (4%) |  |  |  |  |
| > last 3 months | 79 (18%) | 25 (18%) |  |  |  |  |
| Frontline work, *n* (%) |  |  |  | 1.30 | 1 (509) | .254 |
| Frontline | 232 (60%) | 81 (66%) |  |  |  |  |
| Not frontline | 154 (40%) | 42 (34%) |  |  |  |  |
| Changed tasks, *n* (%) |  |  |  | 0.0498 | 1 (575) | .823 |
| Yes | 231 (54%) | 78 (55%) |  |  |  |  |
| No | 201 (47%) | 65 (46%) |  |  |  |  |
| Passive support, *n* (%) |  |  |  | 3.67 | 1 (564) | .056 |
| Participated | 325 (77%) | 117 (84%) |  |  |  |  |
| Not participated | 100 (24%) | 22 (16%) |  |  |  |  |
| Active support, *n* (%) |  |  |  | 4.57 | 1 (367) | .033 |
| Participated | 142 (55%) | 46 (43%) |  |  |  |  |
| Not participated | 117 (45%) | 62 (57%) |  |  |  |  |

**Table S2**

Drop-out analysis at the second follow-up.

|  | Non-dropouts | Dropouts | *t* | χ^2^ | *df* | *p* |
| --- | --- | --- | --- | --- | --- | --- |
| Age, *M* (*SD*) | 46.2 (10.9) | 42.1 (11.6) | -4.21 |  | 571 | <.001 |
| Group, *n* (%) |  |  |  |  |  |  |
| Case | 74 (19%) | 45 (23%) |  | 1.02 | 1 (581) | .313 |
| Control | 310 (81%) | 152 (77%) |  |  |  |  |
| Hospital site, *n* (%) |  |  |  | .0108 | 1 (581) | .917 |
| Site 1 | 127 (33%) | 66 (33%) |  |  |  |  |
| Site 2 | 257 (67%) | 131 (67%) |  |  |  |  |
| Gender, *n* (%) |  |  |  | 0.587 | 1 (577) | .444 |
| Women | 304 (80%) | 151 (77%) |  |  |  |  |
| Men | 77 (20%) | 45 (23%) |  |  |  |  |
| Occupation, *n* (%) |  |  |  | 4.25 | 3 (577) | .236 |
| Assistant Nurse | 83 (22%) | 43 (22%) |  |  |  |  |
| Nurse | 134 (35%) | 84 (43%) |  |  |  |  |
| Physician | 70 (18%) | 32 (16%) |  |  |  |  |
| Non-clinical | 94 (25%) | 37 (19%) |  |  |  |  |
| Tenure, *n* (%) |  |  |  |  |  |  |
| 0-1 years | 9 (2%) | 13 (7%) |  | 12.8 | 3 (564) | .005 |
| 2-5 years | 55 (15%) | 43 (19%) |  |  |  |  |
| 6-10 years | 55 (15%) | 37 (19%) |  |  |  |  |
| 11+ years | 255 (68%) | 107 (56%) |  |  |  |  |
| Mental health history at inclusion, *n* (%) |  |  |  |  |  |  |
| No history | 295 (77%) | 154 (79%) |  | 0.258 | 2 (576) | .879 |
| Last 3 months | 15 (4%) | 8 (4%) |  |  |  |  |
| > Last 3 months | 71 (19%) | 33 (17%) |  |  |  |  |
| Frontline work, *n* (%) |  |  |  | 3.07 | 1 (509) | .080 |
| Frontline | 195 (59%) | 118 (67%) |  |  |  |  |
| Not frontline | 137 (41%) | 59 (33%) |  |  |  |  |
| Changed tasks, *n* (%) |  |  |  | 0.448 | 1 (575) | .503 |
| Yes | 191 (52%) | 208 (55%) |  |  |  |  |
| No | 94 (48%) | 172 (45%) |  |  |  |  |
| Passive support, *n* (%) |  |  |  | 1.54 | 1 (373) | .215 |
| Participated | 213 (86%) | 113 (90%) |  |  |  |  |
| Not participated | 35 (14%) | 12 (10%) |  |  |  |  |
| Active support, *n* (%) |  |  |  | 2.46 | 1 (562) | .117 |
| Participated | 219 (59%) | 97 (52%) |  |  |  |  |
| Not participated | 155 (41%) | 91 (48%) |  |  |  |  |

**Table S3**

Drop-out analysis at the third follow-up.

|  | Non-dropouts | Dropouts | *t* | χ^2^ | *df* | *p* |
| --- | --- | --- | --- | --- | --- | --- |
| Age, *M* (*SD*) | 46.4 (11.0) | 42.8 (11.4) | -3.82 |  | 571 | <.001 |
| Group, *n* (%) |  |  |  |  |  |  |
| Case | 66 (20%) | 53 (21%) |  | 0.0826 | 1 (581) | .774 |
| Control | 263 (80%) | 199 (79%) |  |  |  |  |
| Hospital site, *n* (%) |  |  |  | 0.0925 | 1 (581) | .761 |
| Site 1 | 111 (34%) | 82 (33.5%) |  |  |  |  |
| Site 2 | 218 (66%) | 170 (67.5%) |  |  |  |  |
| Gender, *n* (%) |  |  |  | 1.45 | 1 (577) | .228 |
| Women | 252 (77%) | 201 (81%) |  |  |  |  |
| Men | 75 (23%) | 47 (19%) |  |  |  |  |
| Occupation, *n* (%) |  |  |  | 9.28 | 3 (577) | .026 |
| Assistant Nurse | 62 (19%) | 64 (26%) |  |  |  |  |
| Nurse | 119 (36%) | 99 (40%) |  |  |  |  |
| Physician | 70 (21%) | 32 (13%) |  |  |  |  |
| Non-clinical | 76 (23%) | 55 (22%) |  |  |  |  |
| Tenure, *n* (%) |  |  |  | 9.05 | 3 (564) | .029 |
| 0-1 years | 7 (2%) | 14 (6%) |  |  |  |  |
| 2-5 years | 43 (13%) | 46 (19%) |  |  |  |  |
| 6-10 years | 52 (16%) | 40 (16%) |  |  |  |  |
| 11+ years | 218 (68%) | 144 (59%) |  |  |  |  |
| Mental health history at inclusion, *n* (%) |  |  |  | 1.60 | 2 (576) | .448 |
| No history | 250 (76%) | 199 (80%) |  |  |  |  |
| Last 3 months | 13 (4%) | 10 (4%) |  |  |  |  |
| > last 3 months | 65 (20%) | 39 (16%) |  |  |  |  |
| Frontline work, *n* (%) |  |  |  | 1.02 | 1 (509) | .314 |
| Frontline | 171 (60%) | 142 (64%) |  |  |  |  |
| Not frontline | 116 (40%) | 80 (36%) |  |  |  |  |
| Changed tasks, *n* (%) |  |  |  | 7.47 | 1 (575) | .006 |
| Yes | 159 (49%) | 150 (60%) |  |  |  |  |
| No | 167 (51%) | 99 (40%) |  |  |  |  |
| Passive support, *n* (%) |  |  |  | 0.235 | 1 (564) | .628 |
| Participated | 250 (78%) | 192 (79%) |  |  |  |  |
| Not participated | 72 (22%) | 50 (21%) |  |  |  |  |
| Active support, *n* (%) |  |  |  | 0.0550 | 1 (562) | .815 |
| Participated | 178 (56%) | 138 (57%) |  |  |  |  |
| Not participated | 141 (44%) | 105 (43%) |  |  |  |  |

## RELIABILITY ANALYSIS

**Table S4**

Psychometric properties of the burnout scales.

|  | Mean (SD) | Cronbach’s α | McDonald’s ω |
| --- | --- | --- | --- |
| SMBQ (range 1-7) |  |  |  |
| Follow-up 1 | 2.67 (1.44) | .912 | .915 |
| Follow-up 2 | 2.96 (1.49) | .902 | .907 |
| Follow-up 3 | 3.01 (1.54) | .906 | .909 |
| OLBI (range 1-4) |  |  |  |
| Baseline | 2.39 (0.60) | .733 | .816 |
| Follow-up 1 | 2.20 (0.76) | .901 | .902 |
| Follow-up 2 | 2.40 (0.80) | .915 | .916 |
| Follow-up 3 | 2.41 (0.80) | .919 | .920 |

**Table S5**

Correlation of items in measurement of depressive symptoms.

|  | Mean (SD) | Spearmans’s *Rho* | *p* |
| --- | --- | --- | --- |
| PHQ-2 (range 0-3) |  |  |  |
| Follow-up 1 | 0.58 (0.75) | .611 | < .001 |
| Follow-up 2 | 0.76 (0.82) | .666 | < .001 |
| Follow-up 3 | 0.692 (0.81) | .705 | < .001 |

## RESPONSES ON SECONDARY PREDICTORS

**Table S6**

Proportions of participants in frontline, with changed work tasks and participation in psychological support at each survey.

|  | Baseline  *n* (%) | Follow-up 1  *n* (%) | Follow-up 2  *n* (%) | Follow-up 3  *n* (%) |
| --- | --- | --- | --- | --- |
| Frontline | 313 (62%) | 66 (18%) | 138 (36%) | 59 (18%) |
| Changed tasks | 309 (54%) | 92 (22%) | 95 (25%) | 69 (21%) |
| Passive support | 442 (78%) | 150 (36%) | 292 (83%) | 191 (63%) |
| Active support | 316 (56%) | 151 (37%) | 201 (59%) | 125 (42%) |

## LOGISTIC REGRESSION MODELS

**Burnout as outcome**

Statistical Models 1-3 with burnout as outcome for each follow-up separately, presented in Table S5-S7.

**Table S7**

First follow-up, logistic regression models with burnout as outcome measure.

|  | Model 1 | | Model 2 | | Model 3 | |
| --- | --- | --- | --- | --- | --- | --- |
|  | *OR* | 95% CI | *OR* | 95% CI | *OR* | 95% CI |
| Intercept | 0.184*** | 0.137 - 0.228 | 0.163*** | 0.111 - 0.241 | 0.163*** | 0.104 - 0.254 |
| Case | 3.061*** | 1.798 - 5.211 | 3.120*** | 1.774 - 5.489 | 2.979*** | 1.687 - 5.260 |
| Frontline |  |  | 1.647 | 0.861 - 3.147 | 1.661 | 0.865 - 3.189 |
| Changed tasks |  |  | 1.292 | 0.691 - 2.419 | 1.255 | 0.666 - 2.364 |
| Passive support |  |  |  |  | 1.543 | 0.392 - 6.065 |
| Active support |  |  |  |  | 0.741 | 0.189 - 2.908 |
| Deviance | 396 |  | 342 |  | 348 |  |
| AIC | 400 |  | 350 |  | 350 |  |
| R^2^ McFadden | .039 |  | .049 |  | .048 |  |
| R^2^ Nagel-kerke | .061 |  | .076 |  | .075 |  |

* *p* < .05, ** *p* < .01, *** *p* < .001

**Table S8**

Second follow-up, logistic regression models with burnout as outcome measure.

|  | Model 1 | | Model 2 | | Model 3 | |
| --- | --- | --- | --- | --- | --- | --- |
|  | *OR* | 95% CI | *OR* | 95% CI | *OR* | 95% CI |
| Intercept | 0.230*** | 0.171 - 0.311 | 0.162*** | 0.105 - 0.249 | 0.193*** | 0.093 - 0.402 |
| Case | 5.018*** | 2.868 - 8.780 | 4.594*** | 2.593 - 8.140 | 4.496*** | 2.451 - 8.249 |
| Frontline |  |  | 1.773 | 1.040 - 3.022 | 1.706 | 0.974 - 2.989 |
| Changed tasks |  |  | 1.529 | 0.852 - 2.745 | 1.514 | 0.816 - 2.809 |
| Passive support |  |  |  |  | 0.871 | 0.423 - 1.792 |
| Active support |  |  |  |  | 0.861 | 0.482 - 1.538 |
| Deviance | 368 |  | 354 |  | 325 |  |
| AIC | 372 |  | 362 |  | 337 |  |
| R^2^ McFadden | .080 |  | .093 |  | .089 |  |
| R^2^  Nagelkerke | .128 |  | .149 |  | .140 |  |

* *p* < .05, ** *p* < .01, *** *p* < .001

**Table S9**

Third follow-up, logistic regression models with burnout as outcome measure.

|  | Model 1 | | Model 2 | | Model 3 | |
| --- | --- | --- | --- | --- | --- | --- |
|  | *OR* | 95% CI | *OR* | 95% CI | *OR* | 95% CI |
| Intercept | 0.266*** | 0.195 - 0.363 | 0.206*** | 0.141 - 0.301 | 0.225*** | 0.131 - 0.387 |
| Case | 4.874*** | 2.699 - 8.802 | 4.434*** | 2.383 - 8.250 | 4.622*** | 2.443 - 8.746 |
| Frontline |  |  | 3.357*** | 1.741 - 6.473 | 3.511*** | 1.765 - 6.983 |
| Changed tasks |  |  | 1.091 | 0.557 - 2.137 | 1.041 | 0.515 - 2.105 |
| Passive support |  |  |  |  | 0.816 | 0.437 - 1.525 |
| Active support |  |  |  |  | 1.089 | 0.589 - 2.012 |
| Deviance | 330 |  | 313 |  | 295 |  |
| AIC | 334 |  | 321 |  | 307 |  |
| R^2^ McFadden | .078 |  | .116 |  | .121 |  |
| R^2^  Nagelkerke | .128 |  | .186 |  | .193 |  |

* *p* < .05, ** *p* < .01, *** *p* < .001

**Depressive symptoms as outcome**

Statistical Models 1-3 with depressive symptoms as outcome. For each follow-up separately, presented in Table S8-S10.

**Table S10**

First follow-up, logistic regression models with depressive symptoms as outcome measure.

|  | Model 1 | | Model 2 | | Model 3 | |
| --- | --- | --- | --- | --- | --- | --- |
|  | *OR* | 95% CI | *OR* | 95% CI | *OR* | 95% CI |
| Intercept | 0.139*** | 0.099 - 0.194 | 0.143*** | 0.095 - 0.217 | 0.159*** | 0.100 - 0.253 |
| Case | 3.175*** | 1.795 - 5.615 | 3.051*** | 1.669 - 5.578 | 2.927*** | 1.591 - 5.384 |
| Frontline |  |  | 0.802 | 0.351 - 1.832 | 0.809 | 0.353 - 1.852 |
| Changed tasks |  |  | 1.304 | 0.664 - 2.560 | 1.339 | 0.675 - 2.659 |
| Passive support |  |  |  |  | 0.455 | 0.103 - 2.011 |
| Active support |  |  |  |  | 1.810 | 0.414 - 7.927 |
| Deviance | 342 |  | 298 |  | 294 |  |
| AIC | 346 |  | 306 |  | 306 |  |
| R^2^ McFadden | .042 |  | .044 |  | .046 |  |
| R^2^ Nagel-kerke | .062 |  | .066 |  | .069 |  |

* *p* < .05, ** *p* < .01, *** *p* < .001

**Table S11**

Second follow-up, logistic regression models with depressive symptoms as outcome measure.

|  | Model 1 | | Model 2 | | Model 3 | |
| --- | --- | --- | --- | --- | --- | --- |
|  | *OR* | 95% CI | *OR* | 95% CI | *OR* | 95% CI |
| Intercept | 0.200*** | 0.145 - 0.275 | 0.179*** | 0.117 - 0.276 | 0.277*** | 0.135 - 0.570 |
| Case | 3.415*** | 1.917 - 6.083 | 3.086 *** | 1.710 - 5.570 | 2.794** | 1.478 - 5.280 |
| Frontline |  |  | 1.111 | 0.631 - 1.955 | 1.116 | 0.611 - 2.036 |
| Changed tasks |  |  | 1.452 | 0.795 - 2.651 | 1.468 | 0.770 - 2.797 |
| Passive support |  |  |  |  | 0.537 | 0.259 - 1.110 |
| Active support |  |  |  |  | 1.023 | 0.554 - 1.889 |
| Deviance | 336 |  | 331 |  | 298 |  |
| AIC | 340 |  | 339 |  | 310 |  |
| R^2^ McFadden | .047 |  | .047 |  | .049 |  |
| R^2^ Nagelkerke | .074 |  | .074 |  | .077 |  |

* *p* < .05, ** *p* < .01, *** *p* < .001

**Table S12**

Third follow-up, logistic regression models with depressive symptoms as outcome measure.

|  | Model 1 | | Model 2 | | Model 3 | |
| --- | --- | --- | --- | --- | --- | --- |
|  | *OR* | 95% CI | *OR* | 95% CI | *OR* | 95% CI |
| Intercept | 0.196*** | 0.138 - 0.277 | 0.177*** | 0.118 - 0.265 | 0.162*** | 0.087 - 0.296 |
| Case | 2.808** | 1.502 - 5.250 | 2.657 ** | 1.388 - 5.088 | 2.872** | 1.481 - 5.569 |
| Frontline |  |  | 1.585 | 0.776 - 3.238 | 1.731 | 0.836 - 3.577 |
| Changed tasks |  |  | 1.047 | 0.513 - 2.135 | 0.986 | 0.468 - 2.089 |
| Passive support |  |  |  |  | 1.312 | 0.668 - 2.574 |
| Active support |  |  |  |  | 0.800 | 0.417 - 1.535 |
| Deviance | 288 |  | 282 |  | 267 |  |
| AIC | 292 |  | 290 |  | 279 |  |
| R^2^ McFadden | .034 |  | .040 |  | .050 |  |
| R^2^ Nagelkerke | .053 |  | .062 |  | .078 |  |

* *p* < .05, ** *p* < .01, *** *p* < .001

**References**

1. Appelbom S, Bernhardtz R, Bujacz A. Risk för psykisk ohälsa och behov av psykologiska stödinsatser i samband med covid-19-pandemin: Teknisk rapport [Internet]. Karolinska Institutet; 2023. Available from: https://ki.se/media/259347/download?attachment
